# Physiopathology, Etiologic Factors, Diagnosis, and Course of Polycythemia Vera as Related to Therapy According to William Dameshek, 1940-1950

**DOI:** 10.4274/Tjh.2013.0029

**Published:** 2013-06-05

**Authors:** Jan Jacques Michiels, Goodheart Institute and Foundation, Freedom of Science and Education Thrombocythemia Vera Study Group, TVSG and European Working Groups on Myeloproliferative Neoplasms: EWG.MPN

**Affiliations:** 1 Erasmus University Medical Center, Rotterdam, the Netherlands

**Keywords:** Physiopathology, Etiologic Factors, diagnosis, Polycythemia vera

## Abstract

According to Dameshek, true polycythemia (polycythemia vera: PV) is a chronic myeloproliferative disorder of the total bone marrow without any evidence of invasiveness, in which erythrocytosis, leukocytosis, and thrombocytosis are all simultaneously present. A possible hereditary or transmitted tendency may be present, but actual familial polycythemia is rare. As to the etiology, Dameshek proposed 2 highly speculative possibilities in 1950: the presence of excessive bone marrow stimulation by an unknown factor or factors, and a lack or a diminution in the normal inhibitory factor or factors. Dameshek’s hypothesis was confirmed in 2005 by Vainchenker in France by the discovery of the acquired JAK2V617F mutation as the cause of 3 phenotypes of classical myeloproliferative neoplasms: essential thrombocythemia, PV, and myelofibrosis. The JAK2V617F mutation induces a loss of inhibitory activity of the JH2 pseudokinase part on the JH1 kinase part of Janus kinase 2 (JAK2). This leads to enhanced activity of the normal JH1 kinase activity of JAK2, which makes the mutated hematopoietic stem cells hypersensitive to the hematopoietic growth factors thrombopoietin, erythropoietin, insulin-like growth factor-1, stem cell factor, and granulocyte colony-stimulating factor, resulting in trilinear myeloproliferation. In retrospect, the situation observed by Dameshek where all “stops” to blood production in the bone marrow are pulled in PV is caused by the JAK2V617F mutation.

Dameshek considered PV patients as fundamentally normal and therefore the treatment should be as physiologic as possible. For this reason, a systematic phlebotomy/iron deficiency method of treatment was recommended; the use of radioactive phosphorus is reserved for refractory cases and cases of major thrombosis. If the patient lives long enough and does not succumb to the effects of thrombosis or other complications, the marrow will gradually show signs of diminished activity. The blood smear shows nucleated red cells, increased polychromatophilia, and immature granulocytes of various types. With increasing reduction of erythropoietic tissue, myelofibrosis becomes more of an organized mass of fibrous tissue. There is prominent extramedullary hematopoiesis in the spleen, which becomes extraordinarily large and in some cases occupies almost the entire abdominal cavity. The enlarged spleen is made up largely of metaplastic marrow tissue in primary myeloid metaplasia of the spleen.

**Conflict of interest:**None declared.

## Abstract (tr)

Dameshek’e göre gerçek polistemi (polistemia vera: PV) eş zamanlı eritrositoz, lökositoz ve trombositozun olduğu, yayılma özelliği göstermeyen, tüm kemik iliğini ilgilendiren miyeloproliferatif bir hastalıktır. Olası kalıtsal veya iletilebilme eğilimi olabilir, ancak gerçek ailevi polistemi nadirdir. Etyolojiye yönelik olarak Dameshek 1950’de iki çok tartışmalı olasılık ileri sürmüştür: bilinmeyen faktör veya faktörlerce kemik iliğinin aşırı uyarılması ve normal baskılayıcı faktör veya faktörlerin eksikliği veya olmaması. Dameshek’in hipotezi 2005 yılında Fransa’da Vainchecker tarafından 3 klasik miyeloproliferatif neoplazinin fenotipinin nedeni olarak edinsel JAK2V617F mutasyonunun keşfi ile ispatlandı: esansiyel trombositemi, PV ve miyelofibroz. JAK2V617F mutasyonu Janus kinaz 2 (JAK2)’nin parçası JH1 kinaz üzerinde yer alan JH2 yalancı kinazın baskılayıcı aktivitesinin kaybına neden olmaktadır. Bu durum JAK2’nin normal JH1 kinaz aktivitesinin artışına neden olarak mutasyona uğramış hematopoetik kök hücrelerini trombopoetin, eritropoetin, insulin-benzeri büyüme faktörü-1, kök hücre faktörü ve granülosit koloni uyarıcı faktor gibi hematopoetik büyüme faktörlerine karşı ileri derecede hassaslaştırmak suretiyle her üç seride miyeloproliferasyona neden olmaktadır. Sonuç olarak, Dameshek tarafından gözlemlenen durum PV’da kemik iliğinde kan üretiminini sınırlayan tüm kontrol basamaklarının JAK2V617F mutasyonu ile ortadan kaldırıldığıdır.

Dameshek PV hastalarını özünde normal olarak değerlendirdiğinden tedavinin mümkün olduğunca fizyolojik olması gerekmekteydi. Bu nedenle, sistematik flebotomi/demir eksikliği tedavi metodu önerildi; radyoaktif fosfor kullanımı dirençli ve major trombozu olan hastalara saklandı. Hasta yeterince uzun yaşar ve tromboz etkisi ya da diğer komplikasyonlara bağlı kaybedilmezse, ilik giderek azalan aktivite belirtileri gösterecektir. Kan yaymasında çekirdekli eritrositler, artmış polikromatofili, değişik tiplerde olgunlaşmamış granülositler izlenir. Eritropoetik dokunun gittikçe azalmasıyla, miyelofibroz daha çok organize bir fibröz doku kitlesine dönüşür. Aşırı büyüyen ve bazı olgularda tüm karın boşluğunu dolduran dalakta belirgin ekstramedüller hematopoez vardır. Büyümüş dalak, büyük ölçüde dalağın primer miyeloid metaplazisi alanında yerleşen metastatik kemik iliğinden meydana gelir.

## INTRODUCTION

**Figure 1:**
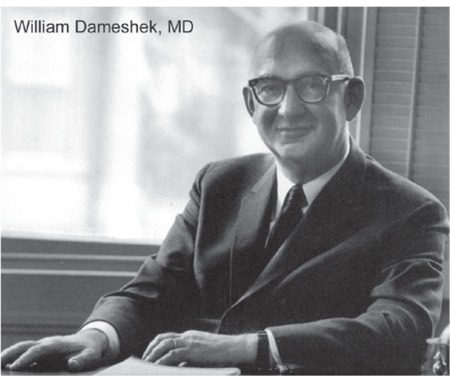
Dameshek (photo courtesy of the American Society of Hematology).

In 1940, Dameshek and Henstell [1] described 20 cases of polycythemia vera (PV) and recognized certain symptoms, signs, and laboratory tests that, when pieced together, spelled out the diagnosis of “primary” or “true” polycythemia. Dameshek (Figure 1) proposed the following groups of symptoms, signs, and laboratory features for the diagnosis of primary polycythemia or PV: 

**Symptoms**

Headache, vertigo, visual disturbances, colored scotoma, acroparesthesias.

Symptoms referable to vascular disturbances of the extremities.

History of profuse hemorrhage after minor trauma.

History of venous and arterial thrombosis.

**Signs**

Plethoric appearance of the face and conjunctivae, dilated retinal veins, splenomegaly, and hepatomegaly.

Red hands and feet and distended capillaries.

**Laboratory Features: Blood and Bone Marrow**

Elevated erythrocyte counts (above 6x10^12^/L), hemoglobin percentage, leukocyte and platelet counts, and hematocrit and polymorphonuclear percentage, together with red cell hyperplasia and megakaryocytic hyperplasia in sternal bone marrow aspirate or biopsy. As in many other rather uncommon diseases, the diagnosis of PV may be overlooked unless it is explicitly considered, and it is likely that many cases are never correctly diagnosed but rather masquerade under the diagnosis of various types of peripheral vascular disease including thromboangiitis obliterans (Burger’s disease), atypical erythromelalgia, or hypertension. Not every symptom, sign, or available bit of laboratory evidence is present in every case of the disease but Dameshek believed in 1940 that the following minimal data should be present before a definite diagnosis of PV can be made: plethoric appearance, splenomegaly, definitely elevated erythrocyte count (>6x 10^12^/L), elevated platelet count, and elevated hematocrit, together with red cell and megakaryocytic hyperplasia in bone marrow aspirate. In a doubtful case, the procedure of blood volume estimation may be helpful.

**Possible Etiologic Factors of PV: Dameshek 1950 [2]**

In 1950, Dameshek wrote a seminal article on the possible etiologic factors, disease manifestations, and course of PV during long-term follow-up, along with his original experiences with treatment by venesection and his hesitations to use radioactive phosphorus (P32). Dameshek considered PV as a disorder of the bone marrow characterized by an excessive production of blood cells by all the marrow elements, i.e. the nucleated red cells, the granulocytes, and the megakaryocytes. To use Greek terms, there is panmyelopathy, which in turn results in pancytosis. The cause of the great and continuous blood production is obscure, but its results lead to many diverse manifestations that, as they are better understood, should lead to better control of the disease.

Little is known of the actual factors that stimulate cell growth. Loss of blood, either through hemorrhage or by excessive destruction (hemolysis), appears to be a powerful stimulatory factor to the bone marrow since the immediate reaction is an outpouring of early red cells (reticulocytes), granulocytes, and platelets. Another stimulatory factor, which, however, affects only the red cells, is that of anoxemia or arterial unsaturation. Whether due to high altitude or to certain types of pulmonary or cardiac disease, there is a resultant increase in erythropoietin. From the teleological standpoint, the increased erythropoietin that takes place with hemorrhage, hemolysis, or anoxemia is readily understood, although the exact mechanisms by which such stimulation takes place in unknown. If stimulatory factors are so little known, even less is known of possible inhibitory factors, although it seems likely that they too are present. Were it not for such “homeostatic” factors, bone marrow growth might conceivably develop out of proportion to the needs of the body. Certain indications are present implicating the spleen as a bone marrow “regulator” or inhibitor, but actual proof of such substances in splenic extracts is thus far lacking. Dameshek concluded in 1950 that although the bone marrow probably has normal stimulants and inhibitors that balance each other, thus resulting in extraordinarily stable blood counts, their nature is obscure.

**Figure 2:**
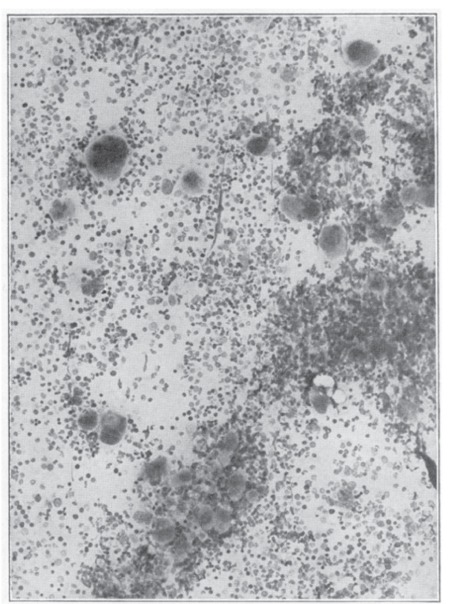
Marked megakaryocytic hyperplasia in the bone marrow of a patient with polycythemia vera. Puncture aspiration of the sternum: 250x. Source: Dameshek, JAMA, 1950 [2].

**Figure 3:**
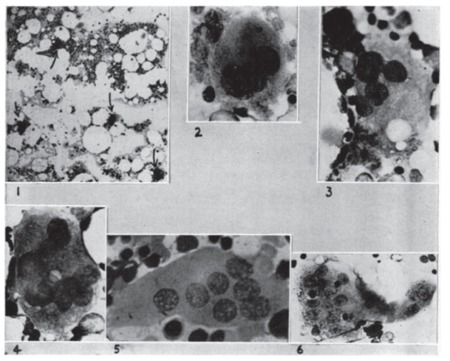
Normal bone marrow: 1) sternal bone marrow puncture smear (75x) showing numerous fat cells, islands of marrow cells, and 4 megakaryocytes (arrows); 2) mature megakaryocyte (750x) with course granularity of the cytoplasm, with defined platelet developments at the edges; 3) and 4) mature megakaryocytes (1000x); 5) polykaryocyte, a multinucleated giant cell, an occasional forerunner of the mature megakaryocyte; 6) polykaryocyte with course granularity and platelet production. Source: Dameshek and Miller, Blood, 1946 [3].

In PV, all “stops” to blood production in the bone marrow seem to have been pulled out. The marrow is crowded with great numbers of nucleated red cells and granulocytes in all stages of maturation and with megakaryocytes actively producing platelets. Because of their large number and their size, the megakaryocytes often dominate the marrow picture (Figure 2). As compared to normal bone marrow (Figure 3), the bone marrow in PV is hypercellular with an increased number of large megakaryocytes (Figure 2).

What causes this enormous productivity of the marrow? Hemorrhage and hemolysis are not present and in fact the hemolytic index in polycythemia may be reduced. There is no evidence of anoxemia; cyanosis is lacking, and the arterial saturation is normal. Some observers have claimed that a local anoxemia affecting only the marrow might be present and have pointed to arteriosclerotic lesions in the marrow as evidence of this. This may well be a mistaken cause-and- effect relationship, and the reverse might be even be true, i.e. the polycythemic state may lead to early arteriosclerosis. Even more importantly, anoxemia leads only to an increase in red cells and not to leukocytosis or thrombocytosis.

Other observers have claimed that polycythemia can be linked to leukemia, i.e. that it is a generalized proliferative disorder involving the red cells. This can hardly be likely, for not only is PV a chronic disorder without any evidence of invasiveness, but it is a total marrow disorder in which erythrocytosis, leukocytosis, and thrombocytosis are all simultaneously present. Neoplastic growth is scarcely like this. Nevertheless, the possibility cannot be ruled out that PV may be a relatively benign, noninvasive, and generalized proliferative disorder of the entire marrow. A possible hereditary or transmitted tendency may be present, since the disorder occurs most commonly in Jews of Russian and Polish origin [4]. Actual familial polycythemia is rare, however. This leaves 2 highly speculative possibilities: the presence of excessive bone marrow stimulation by an unknown factor or factors, and a lack or a diminution in the normal inhibitory factor of factors. Complete ignorance must be admitted at this time of the cause of true polycythemia and of its exact nature. The 1950 hypothesis of Dameshek was confirmed by Vainchenker in France by the discovery in 2005 of the acquired JAK2V617F mutation as the cause of 3 phenotypes of classical myeloproliferative neoplasia:essentialthrombocythemia,PV,andmyelofibrosis [5,6,7,8,9]. The JAK2V617F mutation induces a loss of inhibitory activity of the JH2 pseudokinase part on the JH1 kinase part of Janus kinase 2 (JAK2). This leads to enhanced activity of the normal JH1 kinase activity of JAK2, which makes the mutated hematopoietic stem cells hypersensitive to the hematopoietic growth factors thrombopoietin, erythropoietin, insulin-like growth factor-1, stem cell factor, and granulocyte colony-stimulating factor, resulting in trilinear hypermyeloproliferation. Indeed, all “stops” to blood production in the bone marrow have been pulled [4,5,6,7,8].

**The Disease Polycythemia Vera: Dameshek 1950 [2]**

For whatever reason it is present, panmyelopathy results in an enormous increase in blood cells. All the elements are affected, with the result that the red blood cell count, the polymorphonuclear leukocytes, and the platelets are increased. Individual cases of polycythemia differ greatly, not only from the standpoint of how many blood cells are being produced but also in the relationship of the 3 different marrow elements to each other. For example, some cases show only a moderate elevation in erythrocytes, with, however an extreme degree of thrombocytosis, while in others the leukocytes count may be at or close to leukemic levels, with only slight increase in red blood cells and platelets. The blood counts in PV show the ranges given in Table 1. 

It becomes necessary to define PV further. The normal red cell counts in persons at or near sea level may reach in males the figure of 6,000,000 per cubic millimeter (6x10^12^/L). Certain nervous, high-strung persons with cold acrocyanotic hands and feet or with peptic ulcer or hypertension may habitually show red blood cell values of 6,000,000 to 6,500,000 per cubic millimeter. Persons such as these do not show an associated leukocytosis or thrombocytosis, and the blood volume is uniformly normal; in fact, the plasma volume may even be low, indicating some possible degree of hemoconcentration.

A sharp distinction must furthermore be drawn between true polycythemia and anoxemic or secondary polycythemia. Polycythemia is a panmyelopathy of unknown origin; anoxemic polycythemia is symptomatic of or secondary to various conditions, in which anoxemia is present. The differential clinical and laboratory features are presented in Table 2.

**Figure 4:**
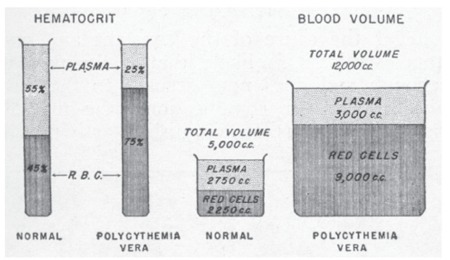
Diagrammatic representation of percentage volume of packed red cells (hematocrit) and blood volume in polycythemia vera as compared to normal. Source: Dameshek, JAMA, 1950 [2].

The most important end result of the extreme degree of panmyelopathy in PV is the development of an enormous mass of red blood cells in the circulation. The percentage volume of packed red blood cells (hematocrit) in relation to the total amount of blood becomes increased greatly, reaching levels of 60% to 70% or even 80%. However, this simple increase in red cell concentration is in itself insufficient to cope with the continued great production of red blood cells (Figure 4). As a result, the total volume of blood is expanded to double or triple the normal values. In parallel with this increased volume, the hematocrit continues to rise such that the total red cell mass, which is normally about 45% of 5000 cc (2250 cc), may finally become increased in some cases to 75% of 15,000 cc (11,250 cc) or to an amount of 4 or 5 times the normal. The plasma volume in such circumstances may remain normal or become slightly increased. In the hypothetic examples given, the plasma volume becomes increased to 3750 cc even though the percentage of plasma is only 25% as compared to the normal percentage of 55%. The great increase in red cell mass leads to an abnormal type of physiologic state and thus to a host of clinical symptoms and signs.

**Physiopathology and Symptoms of PV: Dameshek 1950 [2]**

The presence of a great mass of blood in the circulation has at least 3 outstanding effects: 1) plethora, 2) decreased velocity of blood flow, and 3) increased viscosity of the blood. The person with PV is actually “bursting with blood”. All the various organs become distended with a superabundance of blood, and as a result symptoms referable to all the organs of the body develop. This is particular true of the brain, which is enclosed in the “closed box” of the skull.

The greater the hematocrit level is, the slower the velocity of blood flow is. Slow blood flow and increased blood volume may mimic the symptoms of cardiac decompensation. Slow blood flow may also result in disturbances of the extremities, particularly of the feet. The combined effects of sluggish blood flow, increased viscosity, and the very high platelet level are undoubtedly responsible for the thrombotic manifestations that occur so commonly in PV. Peripheral vascular lesions are most common, but coronary thrombosis, cerebral thrombosis, mesenteric thrombosis, hepatic vein thrombosis (Budd– Chiari syndrome), and even portal vein thrombosis may occur. Thrombotic disturbances of the stomach may be the cause for the rather common finding of gastric ulcer. This was present in about 1 of every 5 of the cases observed by my colleagues and me. The peripheral vascular lesions may simulate those of thromboangiitis obliterans, especially since both polycythemia and thromboangiitis appear most commonly in Russian and Polish Jews.

**Course of PV during Long-Term Follow-Up**

**Figure 5:**
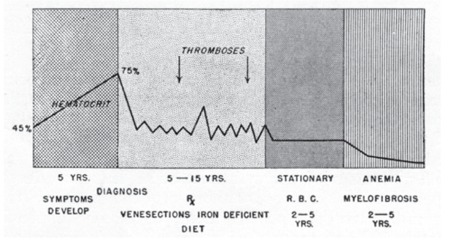
Diagrammatic representation of the course of polycythemia vera in some cases. The marrow becomes “burnt out” and anemia develops in about 2 of 10 cases. Source: Dameshek, JAMA, 1950 [2].

Whatever the cause of the constant and excessive hematopoiesis is, it is likely that if the patient lives long enough and does not succumb to the effects of thrombosis or other complications, the marrow will gradually show signs of diminished activity. If this occurs, the red cell elements become reduced in number and a certain degree of fibrosis develops. With increasing reduction of erythropoietic tissue, myelofibrosis becomes more of an organized mass of fibrous tissue. Leukocyte production, at first unaffected, also becomes reduced, but megakaryocytes usually remain and the lack of other cell types is usually conspicuous. The blood smear shows nucleated red cells, increased polychromatophilia, and immature granulocytes of various types. The continued myelofibrosis either results in or is associated with (it is not clear which) extramedullary hematopoiesis in the spleen and, to a lesser extent, in other organs (Figure 5). The spleen becomes extraordinarily large and in some cases occupies almost the entire abdominal cavity. It is made up largely of metaplastic marrow tissue (myeloid metaplasia).

The course of the patient with PV (Figure 5) is usually a matter of many years, although it is difficult to state what the “normal” course of the disease would be without the various therapeutic methods that undoubtedly influence it.

The cause of the hemorrhagic diathesis of polycythemia is obscure. Bleeding does not ordinarily occur spontaneously, but rather only in response to trauma, as with a blow or following operative procedures. Extreme degrees of postoperative hematomas are common, and excessive bleeding occurs after dental extractions, tonsillectomy, polypectomy, and similar procedures. In view of the high platelet levels, the lack of any well-demonstrated abnormality of the coagulation factors in the blood, and the lack of any apparent abnormality of the capillaries, the cause of hemorrhagic tendency is obscure. In relapse, the bleeding might be explicable on the basis of “bursting with blood”, where an outlet often bleeds severely when the red cell mass has been greatly reduced.

**Treatment of PV, 1940-1950**

The treatment of polycythemia has passed through several successive stages. Phenylhydrazine, a hemolytic poison introduced by Morawitz, achieved wide popularity, but its unpredictable nature and the severe hemolytic crises that often ensued made its use relatively hazardous. All the elements, including iron for enhancing further red cell formation, were retained within the body. For these reasons, the systematic use of multiple venesections to reduce the red cell mass and the blood volume and to induce a state of iron deficiency seemed far more physiologic. According to Dameshek in 1946, the removal of 500 cc of blood twice weekly for 2 to 5 weeks, depending upon the initial hematocrit and hemoglobin levels, has proven to be a satisfactory method. To maintain the resulting state of iron deficiency somewhat longer, it has been our practice to keep the patient on a diet low in iron. Red cell formation under these circumstances is only partially reduced, but due to microcytosis of red cells, hemoglobin and hematocrit levels remain low for periods of 6 to 18 months, during which time the patient may be completely asymptomatic. Red cell levels during this induced remission of PV by phlebotomy alone gradually rise and remain at erythrocythemic levels above 6x10^12^/L, and so the red cell count as an index of therapy is of little value. The best index of therapy is the hematocrit value, although the hemoglobin concentration alone may be used since this correlates fairly closely with the hematocrit level. With this method of therapy, patients go along for many years with little more difficulty than do others in the older age group in which polycythemia occurs.

The treatment of PV is a long-term project and it is best to consider the polycythemia patients as fundamentally normal [2]. As such, they may have a long lifespan and every attempt should be made to keep the treatment as physiologic as possible. Since the fundamental difficulty in polycythemia is the extreme overproduction of blood by the cells of the bone marrow, which leads to an excessive mass of blood within the circulation, the treatment may be based either on a diminution of marrow production or on the removal of the excessive blood from the circulation.

**Figure 6:**
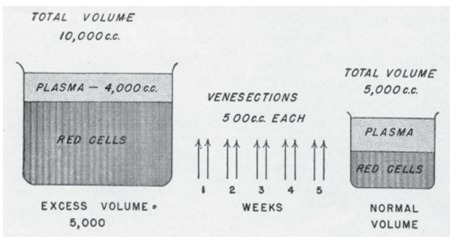
Diagrammatic representation of the plethoric blood volume in a case of polycythemia vera and its correction with systemic venesections performed twice weekly. Source: Dameshek, JAMA, 1950 [2].

**Figure 7:**
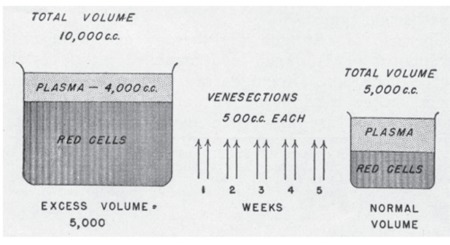
Course in a case of polycythemia vera treated with multiple venesections and iron deficient diet. Note the development of marked microcytic hypochromia: discrepancy between increased red cell count (>6x10^12^/L), diagnostic for PV even in the iron-deficient state, and the relatively low values for hemoglobin and hematocrit. Complete relief of hypervolemic symptoms occurred with this therapy. Source: Dameshek, JAMA, 1950 [2].

The general mode of therapy in polycythemia involves the removal of excessive amounts of blood from the circulation by multiple venesections. The removal of the extra mass of blood by well-planned, multiple venesections is productive of 2 results: 1) the red cell mass, hemoglobin, and hematocrit are reduced to normal, whereas erythrocyte count remains increased above 6x10^12^L; 2) and the bone marrow develops a state of iron deficiency, with the result that erythrocytes produced are poorly hemoglobinized and microcytic. The mature red cells, although they may continue to increase in number, are small and hypochromic and thus greatly reduced in volume. The reduction of the red cell mass to normal levels is carried out by venesections of 500 cc, which are performed twice weekly. Although estimations of the blood volume are valuable, hematocrit determinations are ordinarily sufficient for clinical use (Figure 6). If these are difficult to obtain, hemoglobin estimations are of definite value, although they are by no means as helpful as hematocrit levels. Usually the hemoglobin concentrations parallel roughly the hematocrit readings. The reduction of the hematocrit level to a normal value of about 45% may require from 2 to 4 weeks (i.e. 4 to 8 venesections). With a hematocrit level of approximately 70%, 8 venesections are ordinarily required. At the end of 2 to 4 weeks, longer in some cases, the patient is usually depleted of this extra blood. The considerable loss of blood results in the loss from the body of much of its readily available iron and thus a state of iron deficiency. In polycythemia, the reserve stores of iron are probably not too great because of the continued need for excessive quantities of iron for production of hemoglobin. In any event, the development of a state of chronic iron deficiency is facilitated (Figure 7).

The reduction in iron reserve leads to an insufficient amount of iron for the synthesis of hemoglobin in the developing red cells, and as a result the mature red cells produced are smaller than normal and occupy less room in the circulation than do normal-sized red cells. Production of red cells probably continues in its ordinarily rapid fashion (persistence of increased erythrocyte counts above 6x10^12^/L) (Figure 7), but the insufficiency of iron for the developing red cells results in pronounced hypochromia and microcytosis.

During the state of chronic iron deficiency, the patient himself presents a normal appearance. On this program it is possible to control patients with polycythemia for a few to many years. Some of our patients have been under observation for periods of 10 to 15 years and are in as good of health now as comparable persons of the same age group. The course of arteriosclerotic and diabetic manifestations is not affected. We have seen no greater incidence of the myelofibrotic state than in patients with polycythemia treated by other measures.

The hemoglobin and hematocrit levels remain low for periods of 3 to 18 months or even longer; usually they parallel each other. The red blood cell count after transient correction to normal by venesection, on the other hand, rises gradually after 2 to 3 months and may reach levels of 6,000,000 to 8,000,000 within 6 months or longer (Figure 7). The mean corpuscular volume of red cells becomes reduced to levels of 60 to 65 μm3. The discrepancy between the high red cell count, far above 6x10^12^/L, and low hemoglobin level becomes increasingly more striking. Within 3 to 8 months, or in some cases longer, the hematocrit reaches distinctly increased levels of above 50%, and at this time the patient usually complains of some symptoms previously present. At this point, 2 or 3 venesections may be required to induce further return of the hemoglobin and hematocrit levels to normal (Figure 7). The amount of blood requiring removal at this time is usually relatively small as compared with that removed during the initial relapse.

Dameshek hesitated to use a potentially dangerous radioactive material in an individual with a relatively long lifespan [2] and questioned whether the acute leukemic states that have occurred in some cases are due to the potentially leukemogenic drug P32 or are associated with the natural history of polycythemia. Whether or not the amounts of radioactivity as administered in the ordinary dose of P32 used in the treatment of PV are harmful or productive of leukemia is not known. In my own experience of about 50 reasonably well-followed cases of polycythemia, acute leukemia developed in only 1 (2%) instance without previous X-ray or radioactive phosphorus therapy. Time alone and the comparison of large numbers of well-documented cases in which patients were treated with or without radioactivity should determine whether or not the therapeutic method of Lawrence and Reinhard (personal communications) using radioactive phosphorus is leukemogenic. In my clinic I have restricted the intravenous use of radioactivity to those patients with PV having frequent thromboses in the presence of an extremely high platelet count or to patients with an unusual degree of refractoriness to therapy with venesections. The reduction that occurs in the blood platelets in patients with thrombosis is satisfactory and usually leads to a prolonged remission with amelioration in the thrombotic tendency.

The more recent introduction of radiophosphorus up to 1950 makes therapy a good deal easier and is ordinarily productive of an excellent remission [3]. Erf described in 1946 [10] 25 cases of primary polycythemia treated with P32. Seventeen showed satisfactory hematologic and clinical remissions, 3 showed unsatisfactory remissions, and in 3 the therapy was too recent to warrant the drawing of conclusions. The duration of the remissions was from 6 months to 3 years. One patient died after gastric hemorrhage, and another died after developing leukopenic myeloid leukemia. In Table 1 are listed the collected results in the treatment of PV with radiophosphorus in 138 cases, with satisfactory results in 124 and unsatisfactory results in 5. Of the 5 deaths, 3 were due to leukopenic myeloid leukemia. In 1946 Dameshek hesitated to use a potentially dangerous radioactive material in an individual with a relatively long lifespan. One naturally wonders whether the acute leukemic states that have occurred in some cases are due to the potentially leukemogenic drug or are associated with the polycythemia. The data at present up to 1950 are insufficient to permit statistical analysis at this point. The results of carefully prospective follow-up studies in cases treated with P32 will be awaited with interest.

As a conclusion, in 1950, Dameshek hypothesized that trilinear PV (erythrocythemia, thrombocythemia and granulocythemia) is caused by an unknown stimulatory and/or inhibitory factor of bone marrow hematopoietic progenitor cells. This hypothesis proved to be true by the discovery of the JAK2V617F mutation as the cause of the 3 main phenotypes of the MPNs essential thrombocythemia (ET), PV and myelofibrosis (MF).

**Conflict of Interest Statement**

The authors of this paper have no conflicts of interest, including specific financial interests, relationships, and/or affiliations relevant to the subject matter or materials included.

## Figures and Tables

**Table 1:**
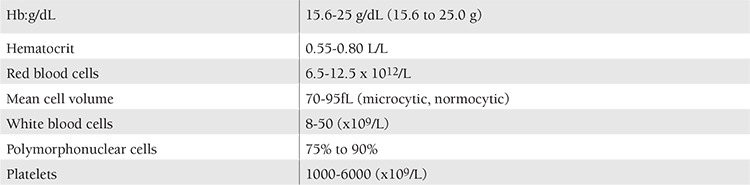
Blood count range in polycythemia vera according to Dameshek, 1950 [2].

**Table 2:**
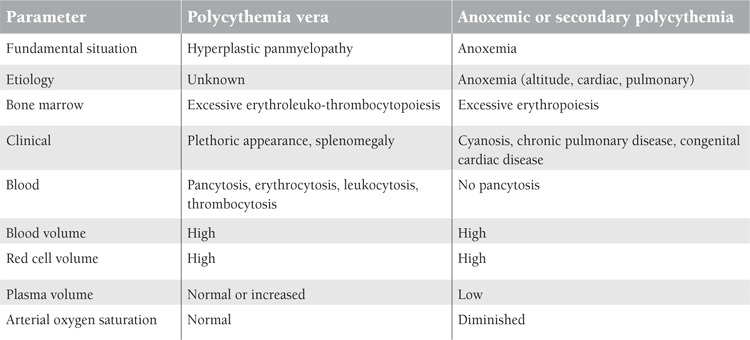
Differential clinical and laboratory features in polycythemia vera and secondary polycythemia according to Dameshek, 1950 [1].

**Table 3:**
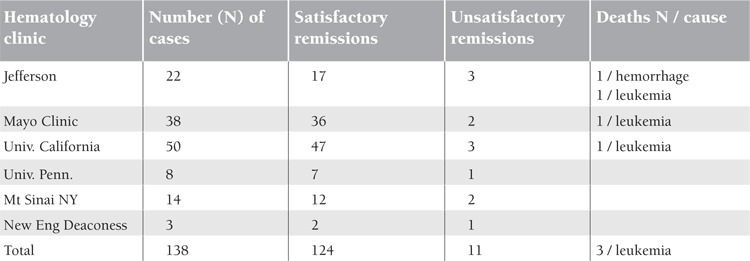
Collected results in the treatment of polycythemia vera with radiophosphorus [10].
